# Characterization of the Synergistic Effect between Ligands of Opioid and Free Fatty Acid Receptors in the Mouse Model of Colitis

**DOI:** 10.3390/molecules26226827

**Published:** 2021-11-11

**Authors:** Agata Binienda, Adam Makaro, Marcin Talar, Julia B. Krajewska, Aleksandra Tarasiuk, Adrian Bartoszek, Adam Fabisiak, Paula Mosińska, Karolina Niewinna, Katarzyna Dziedziczak, Mikołaj Świerczyński, Radzisław Kordek, Maciej Salaga, Jakub Fichna

**Affiliations:** 1 Department of Biochemistry, Faculty of Medicine, Medical University of Lodz, 92-215 Lodz, Poland; agata.binienda@gmail.com (A.B.); adam.makaro@stud.umed.lodz.pl (A.M.); marcin.talar@umed.lodz.pl (M.T.); krajewskajulia@gmail.com (J.B.K.); tarasiuk.aleksandra@gmail.com (A.T.); adrian.bartoszek@stud.umed.lodz.pl (A.B.); adam.fabisiak@umed.lodz.pl (A.F.); paula.mosinska@gmail.com (P.M.); karolina.niewinna@umed.lodz.pl (K.N.); katarzyna.dziedziczak@umed.lodz.pl (K.D.); mikolaj.swierczynski@stud.umed.lodz.pl (M.Ś.); salaga.maciej@gmail.com (M.S.); 2 Department of Digestive Tract Diseases, Medical University of Lodz, 93-281 Lodz, Poland; 3 Department of Pathology, Medical University of Lodz, 92-215 Lodz, Poland; radzislaw.kordek@umed.lodz.pl

**Keywords:** free fatty acid receptors, lipids, opioid receptor, DAMGO, colitis

## Abstract

Background: Recent studies suggest that lipids, including free fatty acids (FFAs), are necessary for proper μ opioid receptor (MOR) binding and that activation of opioid receptors (ORs) improves intestinal inflammation. The objective of the study was to investigate a possible interaction between the ORs and FFA receptors (FFARs) ligands in the colitis. Methods: The potential synergistic effect of ORs and FFARs ligands was evaluated using mouse model of acute colitis induced by dextran sulfate sodium (DSS, 4%). Compounds were injected intraperitoneally (i.p.) once or twice daily at the doses of 0.01 or 0.02 mg/kg body weight (BW) (DAMGO—an MOR agonist), 0.3 mg/kg BW (DPDPE—a δ OR (DOR) agonist) and 1 mg/kg BW (naloxone—a non-selective OR antagonist, GLPG 0974—a FFAR2 antagonist, GSK 137647—a FFAR4 agonist and AH 7614—a FFAR4 antagonist) for 4 days. Results: Myeloperoxidase (MPO) activity was significantly decreased after DAMGO (0.02 mg/kg BW) and GSK 137647 (1 mg/kg BW) administration and co-administration as compared to DSS group. Conclusions: Treatment with ligands of ORs and FFARs may affect the immune cells in the inflammation; however, no significant influence on the severity of colitis and no synergistic effect were observed.

## 1. Introduction

Inflammatory bowel disease (IBD), represented by Crohn’s disease (CD) and ulcerative colitis (UC), is one of the most common gastrointestinal (GI) disorders with unknown etiology. Chronic inflammation, visceral pain, recurrent and alternating diarrhea and constipation are typical symptoms of IBD. Pharmacological treatment includes non-steroid anti-inflammatory drugs (NSAIDs), corticosteroids and biological therapies, e.g., anti-tumor necrosis factor α (anti-TNFα) and anti-α4β7 integrin antibodies. Additionally, surgical procedures, such as resection of the intestine’s inflamed part, may be applied in severe cases [1,2]. Unfortunately, these methods are not fully effective (only about 50% of patients achieve remission), and they may cause serious adverse events (SAEs) [3].

Free fatty acids (FFAs) belong to signaling molecules which act through G protein-coupled receptors (GPCRs) [4]. There are four types of receptors for FFAs: FFAR1 (former nomenclature: GPR40), FFAR2 (GPR43), FFAR3 (GPR41) and FFAR4 (GPR120). FFAR2 and FFAR3 are activated by short chain fatty acids (SCFAs), while FFAR1 and FFAR4 are activated by medium and long chain fatty acids (MCFAs and LCFAs, respectively). FFAR ligands are involved in the regulation of metabolism and in inflammatory processes, also in the gut [5]. Recent studies showed that activation of FFAR1 and FFAR4 might decrease inflammation. In addition, it was showed that FFAR2 ligands are engaged in neutrophil inhibition and subsequently alleviate the severity of inflammation [6,7,8]. These findings suggest that FFARs have the potential to become new pharmacological targets in the treatment of IBD. 

Ligands of the µ opioid receptor (MOR) and δ opioid receptor (DOR), including endogenous opioid peptides, such as β-endorphin and plant-derived opiates, are known for their central and peripheral analgesic effects [9,10]. In line, endogenous opioids were shown to be potentially engaged in pain regulation in chronic IBD [11]. Furthermore, Valdez-Morales et al. [12] proved that the release of endogenous opioids during dextran sulfate sodium (DSS)-induced chronic colitis in mice suppressed the excitability of nociceptive dorsal root ganglia neurons. Interestingly, there is evidence that endogenous opioid peptides are locally produced at the site of inflammation [13]. Consequently, Philippe et al. [14] showed anti-inflammatory properties of MOR agonists in the treatment of colonic inflammation in the mouse models of colitis. Furthermore, DiCello et al. [15] discovered that DOR signaling is enhanced in the enteric nervous system in DSS-induced acute colitis, suggesting that DORs can also be regarded as a potential pharmacological target in IBD treatment.

On the other hand, current studies claim that lipids, including FFAs, are necessary for proper MOR binding [16]. However, the literature on this potentially pharmacologically relevant subject is very scarce. Based on the data summarized above, we hypothesized that the co-administration of opioid receptor (OR) and FFAR ligands may exhibit synergy in alleviation of inflammation and, consequently, influence the course of IBD. Therefore, the aim of the study was to investigate a possible interaction between opioid receptor and FFAR ligands in the mouse model of colitis.

## 2. Results

### 2.1. FFAR2 Antagonist and MOR Agonist Decreased MPO Activity, but Did Not Display Synergistic Anti-Inflammatory Effect

**Figure 1:**
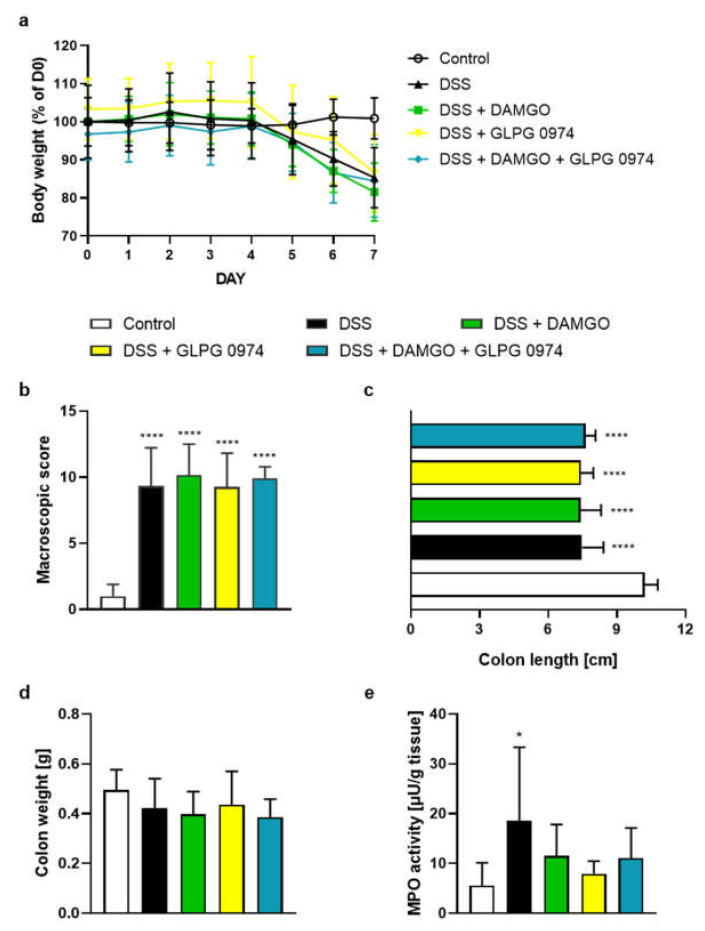
The i.p. administration of MOR agonist DAMGO (0.02 mg/kg BW) and FFAR2 antagonist GLPG 0974 (1 mg/kg BW) from day 3 to day 6, both once daily alleviate MPO activity, marker of DSS-induced colitis in mice. Figure shows data for body weight loss (**a**), macroscopic score (**b**), colon length (**c**), colon weight (**d**) and MPO activity (**e**). * *p* < 0.05, **** *p* < 0.0001, as compared to control mice. Data represent mean ± SEM of 5–10 mice per group. Abbreviations: DSS—dextran sulfate sodium, FFAR4—free fatty acid receptor type 4, i.p.—intraperitoneally, MOR—μ opioid receptor, MPO—myeloperoxidase.

To investigate the possible synergistic anti-inflammatory effect of a MOR agonist DAMGO and a FFAR2 antagonist GLPG 0974 in the mouse GI tract, we used a well-known mouse model of colitis induced by DSS which mimics UC. As shown in Figure 1, animals exposed to DSS developed a severe colonic injury, evidenced by—among others—increased macroscopic damage score and elevated MPO level compared with controls. DAMGO (0.02 mg/kg BW) and GLPG 0974 (1 mg/kg BW), injected once daily did not influence BW loss (Figure 1a), macroscopic score (Figure 1b), colon length (Figure 1c) and colon weight (Figure 1d). However, the co-administration of DAMGO and GLPG 0974 non-significantly decreased MPO activity as compared to DSS group and the compounds alone (Figure 1e).

Bootstrap for hypothesis testing revealed that *P*_bootstrap_ in case of obtained difference in means between DSS + DAMGO vs DSS + DAMGO and GLPG 0974 ligands in macroscopic colon damage score (Figure 1b), colon length (Figure 1c), colon weight (Figure 1d) and MPO activity (Figure 1e) were 0.7928 (calculated absolute difference in means 0.2670), 0.5614 (calculated absolute difference in means 0.2310 cm), 0.7761 (calculated absolute difference in means 0.0132 g) and 0.2105 (calculated absolute difference in means 3.6200 µU/g tissue), respectively.

### 2.2. FFAR4 Antagonist Did Not Influence the Effect of MOR Agonist

**Figure 2:**
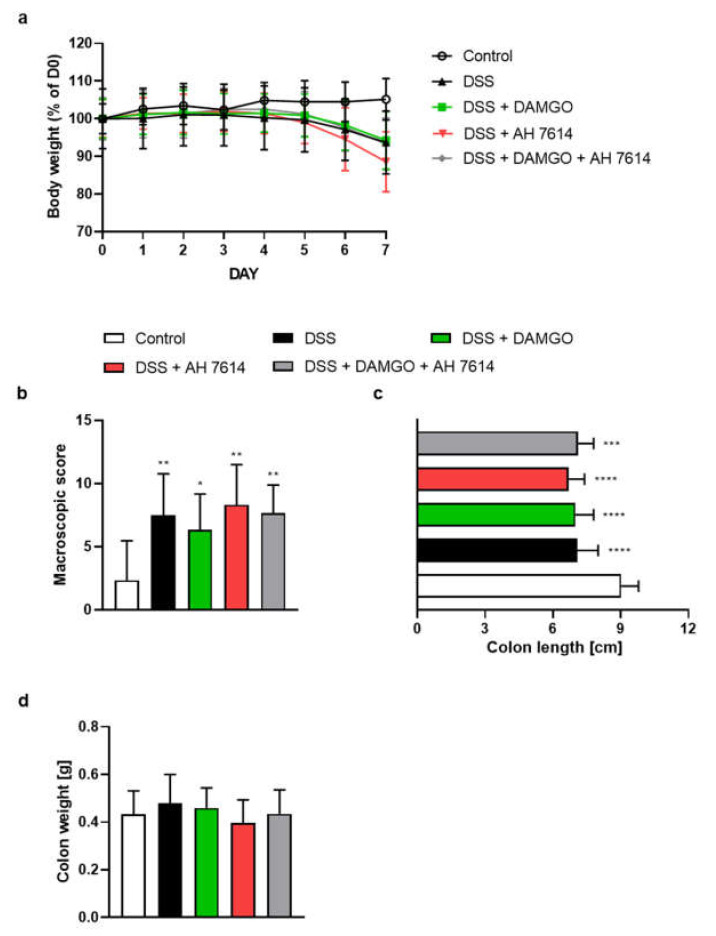
The i.p. administration of MOR agonist DAMGO (0.02 mg/kg BW) and FFAR4 antagonist AH 7614 (1 mg/kg BW) from day 3 to day 6, both twice daily did not alleviate symptoms of DSS-induced colitis in mice. Figure shows data for body weight loss (**a**), macroscopic score (**b**), colon length (**c**) and colon weight (**d**). * *p* < 0.05, ** *p* < 0.01, *** *p* < 0.001 and **** *p* < 0.0001 as compared to control mice. Data represent mean ± SEM of 5–10 mice per group. Abbreviations: DSS—dextran sulfate sodium, FFAR4—free fatty acid receptor type 4, i.p.—intraperitoneally, MOR—μ opioid receptor, MPO–myeloperoxidase.

MOR agonist DAMGO administered twice daily at the dose of 0.02 mg/kg BW (i.p.) and FFAR4 antagonist AH 7614 administered twice daily at the dose of 1 mg/kg BW (i.p.) did not attenuate DSS-induced colitis alone or in co-administration, as indicated by inflammatory indicators (Figure 2a–d).

Bootstrap for hypothesis testing revealed that *P*_bootstrap_ in case of obtained difference in means between DSS + DAMGO vs DSS + DAMGO and AH 7614 ligand in macroscopic colon damage score (Figure 2b), colon length (Figure 2c) and colon weight (Figure 2d) were 0.2445 (calculated absolute difference in means 1.300), 0.6970 (calculated absolute difference in means 0.1240 cm) and 0.5535 (calculated absolute difference in means 0.0239 g), respectively.

### 2.3. FFAR4 Antagonist Did Not Influence the Effect of DOR Agonist

**Figure 3:**
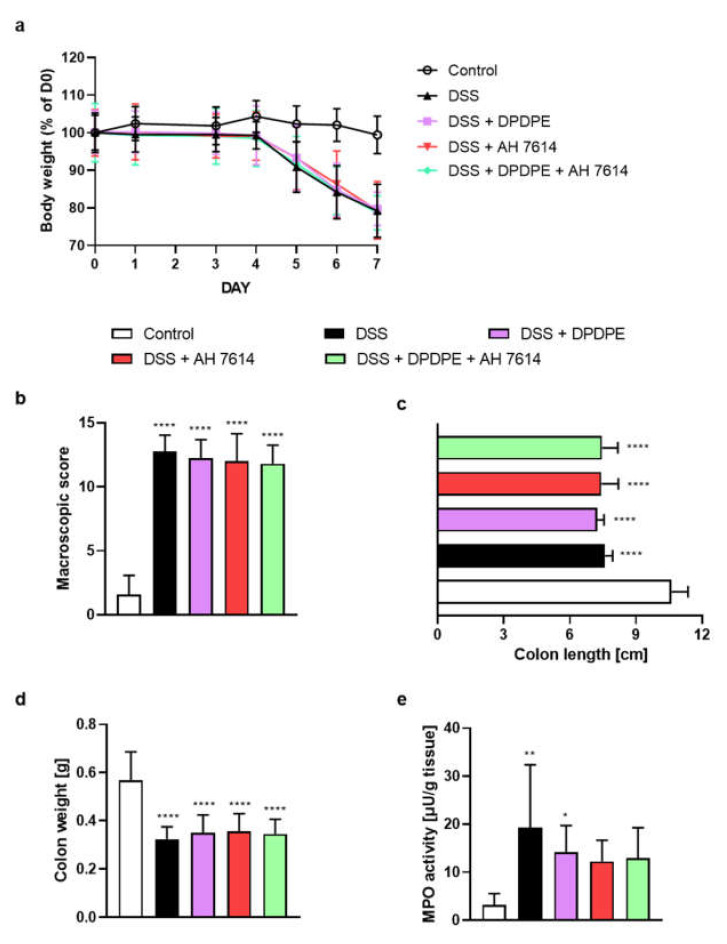
The i.p. administration of DOR agonist DPDPE (0.3 mg/kg BW) and FFAR4 antagonist AH 7614 (1 mg/kg BW) from day 3 to day 6, both twice daily did not alleviate symptoms of DSS-induced colitis in mice. Figure shows data for the body weight loss (**a**), macroscopic score (**b**), colon length (**c**), colon weight (**d**) and MPO activity (**e**). * *p* < 0.05, ** *p* < 0.01 and **** *p* < 0.0001, as compared to control mice. Data represent mean ± SEM of 5–10 mice per group. Abbreviations: DOR—δ opioid receptor, DSS—dextran sulfate sodium, FFAR4—free fatty acid receptor type 4, i.p.—intraperitoneally, MPO—myeloperoxidase.

To test the anti-inflammatory activity of DOR agonist DPDPE and FFAR4 antagonist AH 7614 in the mouse model mimicking UC, a DSS-induced colitis was used. The i.p. administration of DPDPE (0.3 mg/kg BW) and the i.p. administration of AH 7614 (1 mg/kg BW), both twice daily between days 3 and 6 did not induce any improvement in the disease score, as demonstrated by the BW loss (Figure 3a), macroscopic colon damage score (Figure 3b), colon length (Figure 3c), colon weight (Figure 3d) and MPO activity (Figure 3e).

Bootstrap for hypothesis testing revealed that *P*_bootstrap_ in case of obtained difference in means between DSS + DPDPE vs. DSS + DPDPE and AH 7614 ligands in macroscopic colon damage score (Figure 3b), colon length (Figure 3c), colon weight (Figure 3d), and MPO activity (Figure 3e) were 0.8040 (calculated mean absolute difference in means 0.2500), 0.5160 (calculated mean absolute difference in means 0.1800 cm), 0.8918 (calculated mean absolute difference in means 0.0044 g) and 0.6638 (calculated absolute difference in means 1.21 µU/g tissue), respectively.

### 2.4. FFAR4 and MOR Agonists Decreased MPO Activity, but Did Not Display Any Synergistic Effect

**Figure 4:**
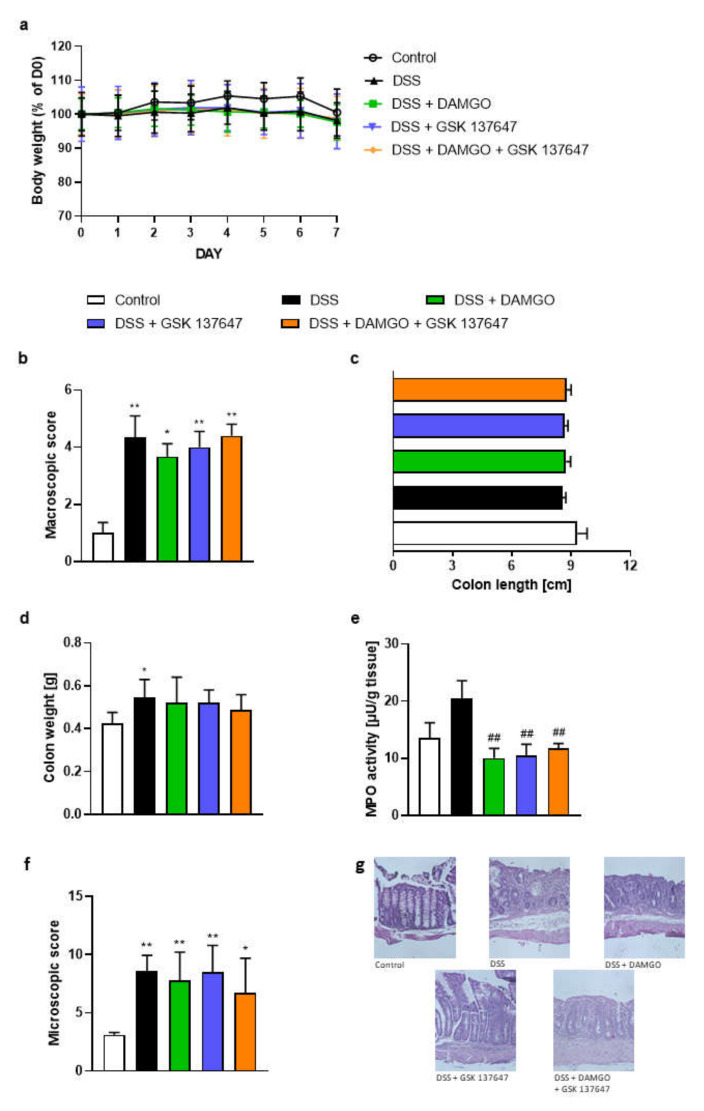
The i.p. administration of MOR agonist DAMGO (0.02 mg/kg BW) and FFAR4 agonist GSK 137647 (1 mg/kg BW) from day 3 to day 6, twice daily alleviated established DSS-induced colitis in mice. Figure shows data for body weight loss (**a**), macroscopic score (**b**), colon length (**c**), colon weight (**d**), MPO activity (**e**) and microscopic score (**f**). Representative photos of hematoxylin and eosin staining of colon samples (**g**). Scale bar = 100 μm. * *p* < 0.05, ** *p* < 0.01 as compared to control mice, whereas ## *p* < 0.01, as compared to DSS-treated mice. Data represent mean ± SEM of 5–10 mice per group. Abbreviations: DSS—dextran sulfate sodium, FFAR4—free fatty acid receptor type 4, i.p.—intraperitoneally, MOR—μ opioid receptor, MPO—myeloperoxidase.

MOR agonist DAMGO at the dose of 0.02 mg/kg BW and FFAR4 agonist GSK 137647 at the dose of 1 mg/kg BW were administered i.p. twice daily from day 3 to day 6. Clinical and macroscopic indicators of colitis, such as BW loss (Figure 4a), macroscopic colon damage score (Figure 4b), colon length (Figure 4c) and colon weight (Figure 4d), were not altered as compared to DSS-treated group.

Noteworthy, the anti-inflammatory effects of DAMGO and GSK 137647 alone and the combination of DAMGO and GSK 137647 were observed on MPO activity (Figure 4e). However, no synergistic effect between MOR and FFAR4 agonists was noted. Moreover, investigated compounds did not influence the microscopic total damage score (Figure 4f,g).

Bootstrap for hypothesis testing revealed that *P*_bootstrap_ in case of obtained difference in means between DSS + DAMGO vs. DSS + DAMGO and GSK 137647 ligands in macroscopic colon damage score (Figure 4b), colon length (Figure 4c), colon weight (Figure 4d), and MPO activity (Figure 4e) were as follows 0.2297 (calculated absolute difference between means 0.7340), 0.8861 (calculated absolute difference in means 0.0441 cm), 0.4470 (calculated absolute difference in means 0.0320 g) and 0.8692 (calculated absolute difference in means 0.0400 µU/g tissue), respectively.

### 2.5. FFAR4 Agonist Did Not Influence the Effect of Opioid Antagonist

**Figure 5:**
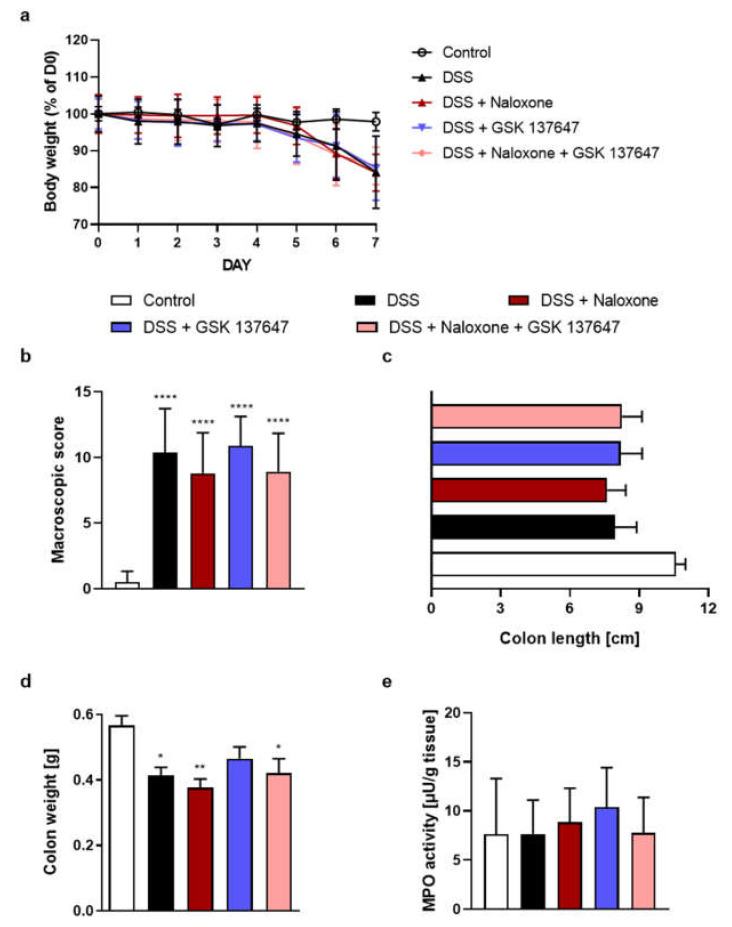
The i.p. administration of opioid receptor antagonist naloxone and FFAR4 agonist GSK 137647 (from day 3 to day 6, both 1 mg/kg BW, twice daily) did not alleviate symptoms of DSS-induced colitis in mice. Figure shows data for body weight loss (**a**), macroscopic score (**b**), colon length (**c**), colon weight (**d**) and MPO activity (**e**). * *p* < 0.05, ** *p* < 0.01 and **** *p* < 0.0001 as compared to control mice. Data represent mean ± SEM of 5–10 mice per group. Abbreviations: DSS—dextran sulfate sodium, FFAR4—free fatty acid receptor type 4, i.p.—intraperitoneally, MPO—myeloperoxidase.

A non-selective opioid receptor antagonist naloxone and FFAR4 agonist GSK 137647, both at the dose 1 mg/kg BW, were administered twice daily from day 3 to 6. The co-administration of naloxone and GSK 137647 in DSS-induced mouse model of colitis did not alleviate symptoms of disease (Figure 5a–e). 

Bootstrap for hypothesis testing revealed that P_bootstrap_ in case of obtained difference in means between DSS + naloxone vs. DSS + naloxone and GSK 137647 ligands in macroscopic colon damage score (Figure 5b), colon length (Figure 5c), colon weight (Figure 5d), and MPO activity (Figure 5e) were 0.1158 (calculated mean absolute difference in means 2.1), 0.1221 (calculated mean absolute difference in means 0.651 cm), 0.3317 (calculated mean absolute difference in means 0.0455 g) and 0.5067 (calculated absolute difference in means 1.1000 µU/g tissue), respectively.

## 3. Discussion

Several lines of evidence suggest the interplay between the endogenous opioid system and FFAs. In the early 1980s, the first reports showed that endogenous opioids contribute to the pathophysiology of central nervous system trauma [17,18]. The opioid mechanism was proved using naloxone, which significantly reversed hypotension and reduced pulse pressure after experimental brain injury. Noteworthy, it was also demonstrated that brain trauma might cause the release of FFAs. In 1990, Bakshi et al. [19] showed that the κ opioid receptor (KOR) agonist (dynorphin)-induced spinal cord tissue damage was connected with the increase in the level of total FFAs, reflecting changes in both saturated and unsaturated fatty acids. It was also evidenced that phospholipid hydrolysis may contribute to this type of injury. In further experiments it was indicated that dynorphin-induced spinal cord tissue damage may be reversed with dynorphin antagonists or non-selective opioid antagonists. Moreover, pretreatment with nalmefene, an opioid receptor antagonist significantly attenuated the increase in total FFAs and individual increases in palmitic acid, stearic acid and oleic acid in spinal cord tissue, providing a potential link between opioids and membrane lipid-dependent mechanism. Finally, Hasegawa et al. [16] suggested that both unsaturated fatty acids and phospholipids can enhance binding of a purified MOR. Additionally, recent studies established that bioactive lipids and MOR cooperate in diabetes [20]. Namely, chronic oral administration of the lipid, 12(*S*)-hydroxyeicosatetraenoic [12(*S*)-HETE] reduced duodenal contractions and improved glucose tolerance. This effect was blocked by the peroxisome proliferator-activated receptor γ (PPARγ) antagonist GW 9662. Since 12(*S*)-HETE is considered as a second messenger that transmits signals from activated MOR, further experiments examined whether MOR is a receptor controlling intestinal contractions and glucose metabolism. The results showed that MOR agonist DAMGO significantly decreased frequency and contraction amplitude of the duodenum of diabetic mice; moreover, this effect was dependent on PPARγ [20].

SCFAs, which modulate FFAR2-dependent pathways, also play an important role in IBD pathophysiology through neutrophil chemotaxis, T cell differentiation and activation and production of cytokines, including tumor necrosis factor α (TNF-α), interleukin (IL)-1β and IL-8 [21,22]. Pizzonero et al. [23] demonstrated that the FFAR2 antagonist GLPG 0974 contributes to inhibition of acetate-induced neutrophil migration and is responsible for reduction of a neutrophil-based pharmacodynamics marker and CD11b activation-specific epitope in a human whole blood assay, suggesting that FFAR2 could be a potential pharmacological target for anti-IBD drugs. Two consecutive randomized, double-blind, placebo-controlled, single-center phase 1 studies (NCT01496937 and NCT01721980) evaluated the safety, tolerability and efficacy of GLPG 0974 in healthy subjects. Results showed that this compound was safe and well-tolerated up to a daily dose of 400 mg [24]. In addition, GLPG 0974 induced substantial and sustained inhibition of acetate-stimulated neutrophil activation. In phase 2 (NCT01829321), patients with mild-to-moderate UC were treated with GLPG 0974 at the dose of 200 mg, twice daily for 28 days. The aim of the study was to examine potential side effects (safety and tolerability) and efficacy of the FFAR2 antagonist. Although reduction of neutrophil activation and infiltration in GLPG 0974-treated individuals were observed, compared to those who received placebo, there were no differences in clinical responses, including Mayo score, or histopathology scoring of colon biopsies.

Recently, the nutritional therapy has begun to play a critical role in IBD course, emphasizing the role of—among others—FFAs in IBD [8,25,26]. Several studies suggest that activation of FFAR4 leads to an anti-inflammatory effect [27,28]. Moreover, patients with IBD have increased expression of FFAR4, which positively correlates with TNF-α level in the gut [29]. However, first reports showed contradictory properties of the selective GPR120/FFAR4 agonist, called compound A in IBD. Oh et al. [30] showed that compound A displays high selectivity and affinity, and is orally available; moreover, it produced potent anti-inflammatory effect on macrophages in vitro and in vivo in obese mice. In contrast, Wannick et al. [31] suggested that oral administration of compound A did not alleviate tissue inflammation in the mouse models of prototypical autoimmune diseases. On the other hand, a synthetic FFAR4 agonist GSK 137647 (administered at the dose of 1 mg/kg BW, i.p., twice daily) alleviated DSS-induced intestinal inflammation in mice, as indicated by significantly reduced MPO activity and macroscopic parameters, such as BW loss [28].

New therapeutics are urgently needed for patients with IBD and current research includes compounds targeting the opioid system [11,13]. Many in vitro and in vivo studies proved that peripherally active opioids decrease the release of pro-inflammatory cytokines or neuropeptides and improve wound healing [21,32,33]. For example, a synthetic MOR agonist DAMGO at the dose of 0.02 mg/kg BW administered in DSS-induced acute colitis significantly decreased disease activity index (DAI) and MPO activity and other parameters of inflammation, such as expression of cytokines and nuclear transcription factor-κB (NF-κB) [34]. Interestingly, lower (0.01 mg/kg BW) and higher (0.04 or 0.08 mg/kg BW) doses of DAMGO did not affect the parameters of inflammation, including DAI and MPO activity suggesting a narrow therapeutic window for the anti-inflammatory properties of this compound [34]. Concurrently, Leanez et al. [35] discovered that a DOR agonist DPDPE demonstrated antinociceptive effect in peripheral inflammation and suggested that it was mainly mediated by nitric oxide derived from nitric oxide synthase 1. DPDPE is also engaged in inhibition of plasma extravasation during chronic intestinal inflammation [36]. Finally, MOR and DOR were found to be expressed by neurons and immune cells in the GI tract, especially in the colon [37] and they have since been considered as attractive pharmacological targets in IBD treatment.

In the present study, the possible synergistic effect of MOR/DOR and FFAR ligands was investigated. Of all the setups examined, the most interesting observation was that the treatment with DAMGO and GSK 137647 alone and in co-administration may affect the immune function in the colonic inflammation through reduction of MPO activity. Furthermore, we evaluated the microscopic total damage to analyze the reduction of neutrophil infiltration in tissues; however, no statistically significant results were obtained. In addition, in our experiments we have not observed any synergistic effects. This may be caused by the fact that no positive, anti-inflammatory effects of DAMGO alone were seen. The probable reason for such outcomes could be the severity of inflammation or doses of tested compounds used in our study (please see Study Limitations below).

To ensure that the revealed mean differences between MOR/DOR ligands alone and in co-administration with FFAR ligands were not observed due to pure chance, we used the bootstrap hypothesis testing technique with 10,000 iterations (see below Equation (1)). The bootstrap method is a statistical procedure that resamples a single dataset to create many simulated samples. Such approach is one of the ways to control and check the stability of the obtained results, especially when we have relatively small sample sizes. It is also a convenient method, often used in life sciences, that avoids the cost of repeating the experiment to obtain other groups of sample data; moreover, it follows 3R recommendations. Hypothesis testing is a fundamental statistical procedure on which all inference is based. It evaluates two mutually exclusive statements about differences in data sets to determine which statement is best supported by the gathered data. We believed that using this technique would support our results and increase the power of our inference.

As indicated above, although DAMGO and GSK 137647 alone and in co-administration decreased MPO activity, when compared to untreated group, we did not observe synergy in their action. On the other hand, the high values of the bootstrap statistics (there were no *P*_bootstrap_ ≤ 0.05) in our experiments indicated that there is still a chance in further experiments of obtaining values of differences between the mean MOR and MOR + FFAR that were greater than or equal to the results we presented. 

## 4. Materials and Methods

### 4.1. Animals

Experimentally naive male BALB/c mice obtained from the vivarium of the University of Lodz, Poland, were used in all experiments. Mice weighed 22–26 g (6–8 weeks of age) and were housed at a constant temperature (22–23 °C) and maintained under a 12-h light/dark cycle (lights on at 6.00 am) in sawdust-lined plastic cages with free access to chow pellets and tap water. All animal protocols were approved by the local Animal Care Committee (Protocols 3/ŁB124/2019 and 11/ŁB128/2019). All efforts were made to minimize animal suffering and to reduce the number of animals used. Groups of 5–10 animals were used in all in vivo experiments.

### 4.2. Drugs

MOR agonist—DAMGO was purchased from Sigma-Aldrich (Poznan, Poland), whereas DOR agonist—DPDPE was obtained from TriMen Chemicals (Lodz, Poland). FFAR ligands, including a FFAR4 agonist—GSK 137647, a FFAR4 antagonist—AH 7614 and a FFAR2 antagonist—GLPG 0974 and naloxone were obtained from Tocris Bioscience (Bristol, UK). DSS (MW 40,000) was purchased from PanReac AppliChem, Lot No.9J013322 (Darmstadt, Germany). All drugs were dissolved in 5% dimethyl sulfoxide (DMSO) in saline, which was used as a vehicle; DSS was dissolved in tap water. The vehicle in the used concentration had no effects on the observed parameters.

### 4.3. Induction of Colitis

Colonic inflammation in this model was induced by DSS (4% wt/vol). DSS was added to drinking water from day 0 to day 5. On days 6 and 7 DSS solution was changed to tap water. Control animals were receiving tap water throughout the whole experiment. Animal body weight and general health and disease progression were monitored daily.

### 4.4. Pharmacological Treatments

In the DSS model, mice were treated with various combinations of opioid receptor and FFAR ligands, namely: DAMGO (0.02 mg/kg BW, intraperitoneally (i.p.), once or twice daily), DPDPE (0.3 mg/kg BW, i.p., twice daily), naloxone (1 mg/kg BW, i.p., twice daily), GLPG 0974 (1 mg/kg BW, i.p., once daily), GSK 137647 (1 mg/kg BW, i.p., twice daily) and AH 7614 (1 mg/kg BW, i.p., twice daily), from day 3 to day 6. On day 7, animals were sacrificed, and evaluation of colonic damage was performed. In all experiments control animals received vehicle (100 μL, i.p.) alone. The doses of opioid receptor and FFAR ligands used in this study were selected based on literature and preliminary studies.

### 4.5. Evaluation of Colonic Damage

Disease parameters were evaluated on day 7 of the experiment. After euthanasia, the entire colon was isolated and weighed with fecal content; colon length was measured using a caliper. Then, the colon was opened longitudinally and cleaned from the fecal content. A total macroscopic damage score was calculated for each animal based on the (i) stool consistency (where 0 means normal well-shaped fecal pellets and 3 means diarrhea), (ii) colon epithelial damage based on a number of ulcers (0–3), (iii) colon length and weight scores expressed as a percentage change of each parameter in relation to the control group (0 points, ≤5% change; 1 point, 5–14% change; 2 points, 15–24% change; 3 points, 25–35% change; and 4 points, ≥35% change). The presence (score = 1) or absence (score = 0) of fecal blood was also recorded. Total score = 0 means no inflammation.

### 4.6. Determination of Tissue Myeloperoxidase Activity

Myeloperoxidase (MPO) activity was quantified with the method described earlier by Salaga et al. [38]. In brief, 0.3 cm segments of colon were weighed and rapidly homogenized in hexadecyltrimethylammonium bromide (HTAB) buffer (0.5% HTAB in 50 mM potassium phosphate buffer, pH 6.0; approx. 30 mg of tissue/mL). Then, the homogenate was centrifuged for 15 min (13,200 *g*, 4 °C) and the supernatant was used in the subsequent steps. On a 96-well plate, 200 μL of 50 mM potassium phosphate buffer (pH 6.0), containing 0.167 mg/mL of *O*-dianisidine hydrochloride and 0.05 μL of 1% hydrogen peroxide was added to 7 μL of the supernatant. Absorbance was measured (in triplicate) at 450 nm (iMARK Microplate Reader, Biorad, United Kingdom) at 0, 30 and 60 s after initiation of reaction. MPO was expressed in milliunits per gram of wet tissue, 1 unit being the quantity of enzyme able to convert 1 μmol of hydrogen peroxide to water in 1 min at room temperature. Units of MPO activity per 1 min were calculated from a standard curve prepared with the use of purified peroxidase enzyme.

### 4.7. Histology 

Segments of the distal colon were stapled flat, mucosal side up, onto cardboard and fixed in 10% neutral-buffered formalin for 24 h at 4 °C. Then, samples were dehydrated in sucrose, embedded in paraffin, sectioned at 5 μm and mounted onto slides. Subsequently sections were stained with hematoxylin and eosin and examined using (Axio Imager A2 microscope, Carl Zeiss, Berlin, Germany). Microscopic total damage score was determined based on the following parameters: presence (score = 1) or absence (score = 0) of goblet cell depletion, the presence (score = 1) or absence (score = 0) of crypt abscesses, the destruction of mucosal architecture (normal = 1, moderate = 2, extensive = 3), the extent of muscle thickening (normal = 1, moderate = 2, extensive = 3) and the presence and degree of immune cell infiltration (normal = 1, moderate = 2, transmural = 3).

### 4.8. Bootstrap for Hypothesis Testing in R Software

To compare numeric variables for two groups/datasets, bootstrap approach to hypothesis testing was implemented. This approach was taken as an alternative to the two-sided t-test for two samples comparing independent groups (all datasets had normal distribution). All calculations were made in RStudio software with utilizing R programing language. Specific code for all computations was written for this purpose, according to guidelines from Marinstats lectures from the University of British Columbia (https://statslectures.com/r-scripts-datasets, accessed on 1 April 2021). Program code was attached in Supplementary Materials. Calculation of the absolute difference in means for MOR vs. MOR + FFAR ligand or DOR vs. DOR + FFAR ligand in colitis was the first step in our approach (observed_test_stat). Next, the matrix of 10,000 bootstrap resamples with replacement from our datasets were made. Each pair of data (column) was a single bootstrap sample. For each of such new random resampled column, the absolute difference in means for MOR vs. MOR + FFAR ligand or DOR vs. DOR + FFAR ligand was calculated through loop (bootstrap_test_stat [*n*, *n* + 1, *n* = 10,000]). Eventually, *P* value of bootstrap (*P*_bootstrap_) was calculated as follows (Equation (1)):

(1) $$P_{\mathrm{bootstrap}}=\frac{{\mathrm{bootstrap}}_{{\mathrm{test}}_{\mathrm{stat}1}}\geq {\mathrm{observed}\_\mathrm{test}\_\mathrm{stat}+\ldots +\mathrm{bootstrap}}_{{\mathrm{test}}_{\mathrm{stat}10000}}\geq \mathrm{observed}\_\mathrm{test}\_\mathrm{stat}}{10000}$$

where *P* value denotes the number of the bootstrap test statistics that were greater than or equal to the observed test statistic divided by the total number of bootstrap test statistics. For example, *P*_bootstrap_ = 0.25 (for calculated absolute difference in means = 13 units) was interpreted as out of the 10,000 bootstrap test statistics calculated, 2500 (25%) of them had test statistic greater than 13 units. This, in turn, was interpreted as follows: if there was no difference between two groups, we would see a test statistic equal or higher than the value of observed_test_stat (absolute difference in the sample means; in the example above: 13) by chance 25% of the time. More generically *p*-values of bootstrap have told us what the probability is of getting the test statistic we got, or if the null hypothesis is true (there is no difference between two groups/datasets). 

### 4.9. Statistics

Statistical analysis was performed using Prism 9.0.1 (GraphPad Software Inc., La Jolla, CA, USA) and RStudio software. The data are expressed as means ± SEM. The Shapiro–Wilk test was used to test the normality of data distribution. One-way analysis of variance (ANOVA) followed by Bonferroni post-hoc test and student t-test were used for analysis. To better evaluate the synergy of the opioid receptor and FFAR ligands, mainly due to relatively small sample sizes and the low statistical power of the estimated inferences, we employed the bootstrap hypothesis testing technique (10,000 iterations) to ensure that the revealed differences in means between MOR vs. MOR + FFAR ligand or DOR vs. DOR + FFAR ligand, in colitis were not observed due to pure chance. *P* values and *P* value of bootstrap (*P*_bootstrap_) < 0.05 were considered as statistically significant.

## 5. Conclusions

Our results showed that treatment with ligands of MOR and FFAR separately and in combination may affect immune function in the inflammation seen by altering MPO activity even though these ligands had no significant influence on the severity of colitis. However, MOR/DOR and FFAR ligands did not exhibit synergistic effect in colonic inflammation, including immune aspect. This study indicates that the interaction between the endogenous opioid system and FFARs, although suggested by some reports, does not take place in colonic inflammation. Nevertheless, there are some premises to continue this research; one of them is the high values of *P*_bootstrap_ statistics in the conducted experiments. Further validation of this interaction as a potential therapeutic target in IBD is thus needed. One of the necessary future experiments should include higher doses of compounds (e.g., DAMGO). Moreover, a new possible interactions should be tested, e.g., for FFAR and hormone binding sites.

## 6. Study Limitations

Although the doses of opioid receptors and FFARs ligands used in our study were selected based on literature [14,29,35] and our preliminary results, we did not obtain any beneficial impact on mouse colitis. Moreover, we concluded the study by investigating whether the co-administration of opioid antagonist naloxone and FFAR4 agonist GSK 137647 may influence parameters of colitis in mice. This opposite direction of our research also did not show any differences between treated and un-treated groups. In line, bootstrap hypothesis testing for DOR ligand and DOR + FFAR ligands revealed the same trend of randomness as mentioned in case of MOR ligand and MOR + FFAR ligands.

## Data Availability

The data presented in this study are available on request from the corresponding author.

## Supplementary Materials

Code Program S1: Bootstrap hypothesis test.

## Sample Availability

Not available.
