# Supplementary material for: Characterization of the Synergistic Effect between Ligands of Opioid and Free Fatty Acid Receptors in the Mouse Model of Colitis
Source: Molecules. 2021 Nov 11;26(22):6827. doi: 10.3390/molecules26226827 (PMC8626023; doi:10.3390/molecules26226827)
Supplement: Supplementary file 1 [file molecules-26-06827-s001.zip › molecules-1400399-supplementary.pdf]

Supplementary Material

S1

```
#### BOOTSTRAP HYPOTHESIS TEST ###

# Script comes from https://statslectures.com/r-scripts-datasets and was modified by M.Talar

#loading data

zmienna = read.csv(file.choose(), header = T, sep = ";")

View(zmienna)

# check the names, etc

names(zmienna)

# how many observations?

table(zmienna$Var)

levels(zmienna$Var)

# Box-plot

boxplot(zmienna$Val~zmienna$Var, las=1, ylab="", xlab="", main="BOX-PLOT: Median + IRQ")

# Mean calculation

mean(zmienna$Val[zmienna$Var=="Group A"])

# Mean calculation /Function/

with(zmienna, tapply(Val, Var, mean))

test.stat1 = abs(diff(with(zmienna, tapply(Val, Var, mean))))

test.stat1

#Median calculation

with(zmienna, tapply(Val, Var, median))

test.stat2 = abs(diff(with(zmienna, tapply(Val, Var, median))))

test.stat2

# 2-sample t-test

t.test(zmienna$Val~zmienna$Var, paired=F, var.eq=F)  # tests Ho: means are equal

# Mann-Whitney U test
```

```
wilcox.test(zmienna$Val~zmienna$Var, paired=F)  # tests Ho: medians are equal

# Kolmogorov-Smirnov 2-sample test

ks.test(zmienna$Val[zmienna$Var=="Group A"],
zmienna$Val[zmienna$Var=="Group B"], paired=F)     # tests Ho: distributions are same

#######################

##  BOOTSTRAPPING... ###

#######################

set.seed(112358)   # The same seed the same results

n = length(zmienna$Var)  # the number of observations to sample

n

l_A = length(zmienna$Val[zmienna$Var == "Group A"])

l_A

seq(l_A)

l_B = length(zmienna$Val[zmienna$Var == "Group B"])

l_B

l_C = l_A + l_B

l_C

l_Cx = l_A + 1

sekB = seq(l_Cx,l_C)

sekB

sekA= seq(1,l_A)

sekA

B = 10000 # the number of bootstrap samples

variable = zmienna$Val  # the variable we will resample from

# now, get those bootstrap samples (without loops!)

BootstrapSamples = matrix( sample(variable, size= n*B, replace=TRUE),

                     nrow=n, ncol=B)

# let's take a moment to discuss what that code is doing...

dim(BootstrapSamples)
```

```
# now, calculate the means (YGroup_A and YGroupB) for each of the bootstrap samples

#  (the inefficeint, but transparent way...best to start simple, and once

#   working well, then make code more efficent)

# initialize the vector to store the TEST-STATS

Boot.test.stat1 <- rep(0,B)

Boot.test.stat2 <- rep(0,B)

# run through a loop, each time calculating the bootstrap test.stat

#  NOTE: could make this faster by writing a "function" and then

#       using "apply" to apply it to columns of the "BootSamples"

for (i in 1:B){

  # calculate the boot-test-stat1 and save it

  Boot.test.stat1[i] <- abs( mean(BootstrapSamples[sekA,i]) -

                          mean(BootstrapSamples[sekB,i]) )

  # calculate the boot-test-stat2 and save it

  Boot.test.stat2[i] <- abs( median(BootstrapSamples[sekA,i]) -

                          median(BootstrapSamples[sekB,i])  )

}

# let's remind ourselves of the OBSERVED TEST STATS

test.stat1; test.stat2

# and, take a look at the first 20 Bootstrap-TEST STATS for 1 and 2

round(Boot.test.stat1[1:50], 1)

round(Boot.test.stat2[1:50], 1)

# and, let's calculate the bootstrap p-value...

# notice how we can ask R a true/false question...(for the first 20)

(Boot.test.stat1 >= test.stat1)[1:20]

# and if we ask for the mean of all of those, it treats 0=FALSE, 1=TRUE

#...calculate the p-value

mean( Boot.test.stat1 >= test.stat1)

#...calculate the p-value for outcomes different than 0

mean( Boot.test.stat1 >0)
```

```
#...calculate the p-value for outcomes equal or less than 0

mean( Boot.test.stat1 <=0)

# let's calculate the p-value for test statistic 2 (abs diff in medians)

mean( Boot.test.stat2 >= test.stat2)

# let's calculate the p-value for test statistic 2 (abs diff in medians >0)

mean( Boot.test.stat2 > 0)

# now, recall the difference between "statistical significance" and

# "scientific significance"

### in a "real-world" what would you want to conclude here

table(zmienna$Var)

# let's take a look at a density plot of all the Bootstrap test-stats, and

# add in our Observed test stat

plot(density(Boot.test.stat1),

    xlab=expression( group("|", bar(Group_A) - bar(Group_B), "|") ) ,

    main="Bootstrap Test Stats", las=1)

abline(v=test.stat1, col="blue", lty="dotted")

text(60,0.0005, "p-value", col="blue", cex=0.7)

##########################

### Code to run the analysis, using a test stat of diff in 90th percentiles

##########################

# lets calculate the absolute diff in 90th percentiles

test.stat3 <- abs(quantile(zmienna$Val[zmienna$Var=="Group A"], prob=0.9) -
quantile(zmienna$Val[zmienna$Var=="Group B"], prob=0.9))  #diff in medians

test.stat3

# initialize a vector to save the bootstrap test stats in

Boot.test.stat3 <- rep(0,B)

# run thru a loop calculating the bootstrap test statistics

for (i in 1:B){

  # calculate the boot-test-stat3 and save it

  Boot.test.stat3[i] <- abs( quantile(BootstrapSamples[sekA,i], prob=0.9) -

                     quantile(BootstrapSamples[sekB,i], prob=0.9) )
```

```
}

# and, calculate the p-value

mean( Boot.test.stat3 >= test.stat3)
```
